# Demonstration of anatomical and technical details of robotic laparoscopic radical prostatectomy as described in the current literature

**DOI:** 10.3389/fsurg.2026.1804051

**Published:** 2026-05-07

**Authors:** J. Rassweiler, Sara Sander, Marie-Claire Rassweiler-Seyfried

**Affiliations:** 1 Department of Urology and Andrology, Danube Private University, Krems, Austria; 2 Department of Urology, University of Tübingen, Tübingen, Germany; 3 Department of Urology, Klinikum Mannheim, University of Mannheim, Mannheim, Germany

**Keywords:** continence, positive margins, robot-assisted laparoscopic radical prostatectomy, surgical technique, video-anatomy

## Abstract

**Background:**

Since robot-assisted laparoscopic prostatectomy (RALP) has nowadays become widely accepted world-wide, there needs to be an agreement on the most efficient surgical techniques. This should be based on the video-anatomy of the prostate and a summary of the actual literature.

**Material and methods:**

Using video material taken during RALP-procedures performed by the same surgeon (J.R.), the anatomical details of different surgical techniques and operative steps are shown based on the anatomy of the male pelvis applying the standard nomenclature. This was supplemented by a systematic literature search in PubMed focusing on preservation of continence and minimal rates of positive margins. 3,825 publications could be reduced to 604 articles according to the inclusion criteria (randomized controlled trials, meta-analyses, systematic reviews and clinical studies), When expanding the search to encompass individual operation techniques, we identified 27 relevant articles.

**Results:**

Crucial surgical details include preserving the levator fascia, the puboprostatic collar, a long urethral stump with protection of the urethral lissosphincter and posteriorly reconstruction of the rectourethralis with the prostatovesical muscle. Fascial preservation for the M. Levator ani results to one year-continence between 78,0 and 98,3%, preservation of the puboprostatic collar and detrusor apron between 95,6 and 100%, maximal functional urethral length between 90,5 and 97,5%. Posterior reconstruction leads to a 3-months continence between 92,3 and 96,9%. Preserving the Retzius` space and thus the total anterior sphincter apparatus results to a one-year continence of 95,8%, however associated with a higher rate of positive surgical margins (14–42 vs. 10–29%).

**Conclusions:**

The increase of knowledge of the video-anatomy of the prostate and surrounding structures allows translation into novel surgical techniques of RALP. Thereby continence rates could be significantly improved including approaches to spare anatomical structures of the sphincter apparatus, such as preservation of the levator fascia, the puboprostatic collar, the urethral lissosphincter, but also reconstructive techniques, such as posterior reconstruction of the vesico-prostatic and recto-urethralis muscle. Demanding techniques, such as the Retzius-sparing approach result to higher continence rates, but are also associated with a higher rate of surgical margins.

## 1 Introduction

As the most common tumour in men, the relevance of prostate cancer is undeniable. Nowadays, the majority of prostate tumours are detected at a locally confined stage due to early detection campaigns (1). The therapeutic options depend on the stage and risk profile of the tumour. For localized prostate cancer, these include active surveillance, radiation therapy, and radical prostatectomy (2, 3). However, the choice of therapy is also crucially influenced by the treatment's side effects, with incontinence and impotence being particularly prominent (1, 2).

Advances in surgical approaches and the introduction of minimally invasive techniques, especially robotic surgery, have significantly reduced the rate of side effects (4). Recently, the superiority of robotic surgery over conventional laparoscopic radical prostatectomy regarding continence has been demonstrated even in the early phase of the learning curve in the LAP-01 trial (5). Furthermore, the use of video-technology has led to an anatomical understanding that is crucial for the success of the operation (6, 7). Additionally, individual patient-specific factors such as age, body mass index (BMI), length of the membranous urethra, or the tumour's location must also be considered (8).

Since numerous modifications of surgical techniques have been presented in the literature, they must be evaluated based on long-term results. It is essential to analyse which surgical steps truly lead to a significant improvement in surgical outcomes. Furthermore, this article should be educational for the reader to understand and possibly adapt certain operative steps when performing robot-assisted laparoscopic radical prostatectomy.

## 2 Materials and methods

### 2.1 Demonstration of video-anatomy of the pelvis

Using video material taken during robotic assisted laparoscopic radical prostatectomies (RALP) performed by the same surgeon (J.R.), the anatomical details of different surgical techniques and operative steps are shown based on the anatomy of the male pelvis applying the standard nomenclature. The terminology was based on classical anatomical and commonly used nomenclature among surgeons (9, 12–28) (Table 1). Furthermore, three articles were included describing the “Heilbronn technique” and its results at different points in time (9–11). The figures taken from these videos have been modified to explain the important anatomical details. All patients have given informed consent to publication.

**Table 1:** Summary of the different descriptions of anatomical details relevant for radical prostatectomy.

Anatomical structure (surgical nomenclature)	Anatomical Desription (anatomical nomenclature) (13)	Eponymic Description	Other Descriptions	Authors
Pelvic fascia (14)	Fascia pelvis parietalis (13)	Endopelvic fascia (16)	-	Walsh (14) Myers (15) Stolzenburg (16)
Levator fascia (14)	Fascia levatoris (13)	Outer layer of „Walsh´s lateral pelvic fascia“(15)	Periprostatic fascia (16) Parapelvic fascia (17)	Walsh (14) Myers (15) Stolzenburg (16) Graefen (17)
Prostate fascia (14)	Fascia prostatica (13)	Innere Schicht der “Walsh´s lateral pelvic fascia” (15)	-	Walsh (14) Myers (15)
Posterior visceral fascia (16)	Septum rectovesicale (13)	Denonvillers` fascia (19)	Prostato-seminal vesicular fascia (18)	Walz (18) Walsh (14) Myers (15) Denonvillers (19) Gil-Vernet (20)
Puboprostatic ligaments (14)	Ligamenta puboprostatica (13)	-	Pubovesical ligaments (15)	Walsh (14) Myers (15)
Dorsal vein plexus (14, 15)	Plexus venosus dorsalis penis (13)	Santorinís plexus (21)	Dorsal vascular complex	Walsh (14) Myers (15) Santorini (21)
Fascial pelvic tendon arc (18)	Arcus tendineus fasciae pelvis (13)	-	Puboprostatic collar (20)	Walz (18) Takenada (22)
Anterior visceral fascia (18)	Fascia pelvis visceralis (13)	-	Anterior periprostatic Fascia (over the Detrusor Apron and McNeal`s anterior fibro-muscular stroma) (14)	Walz (18) Walsh (14) Myers (15) McNeal (23)
Bladder sphincter (18)	M. sphincter vesicalis (13)	Internal sphincter	Smooth muscle Bladder sphincter (18)	Walz (18)
Rhabdosphincter of urethra (14, 15)	M. sphincter urethrae externus (13)	External sphincter	Striated muscle horse-shoe-shaped urethral sphincter	Walsh (14) Myers (15) Walz (18)
Lissosphincter of urethra (14, 15)	M. sphincter urethrae internus (13)	-	Urethral Lissosphincter	Walsh (14) Myers (15) Walz (18)
Mm. puboperineales (24)	M. levator urethrae (Hiatus urogenitalis) (13)	Quick-stop muscle of Gosling (25)	Puboperineal muscle (21)	Walz (18) Dorschner (24) Takenada (22) Gosling (25)
Vesico-prostatic musclel (18, 24)	M. vesico-prostaticus (13)	-	Inner layer of Denonvillers` fascia (20) Posterior longitudinal fascia	Walz (18) Gil-Vernet (20) Dorschner (24) Takenada (22)
Posterior raphe (14, 15)	M. anoperinealis	-	Recto-urethralis muscle (18, 24)	Walsh (14) Myers (15) Walz (18) Dorschner (24)

### 2.2 Literature search

**Figure 1:**
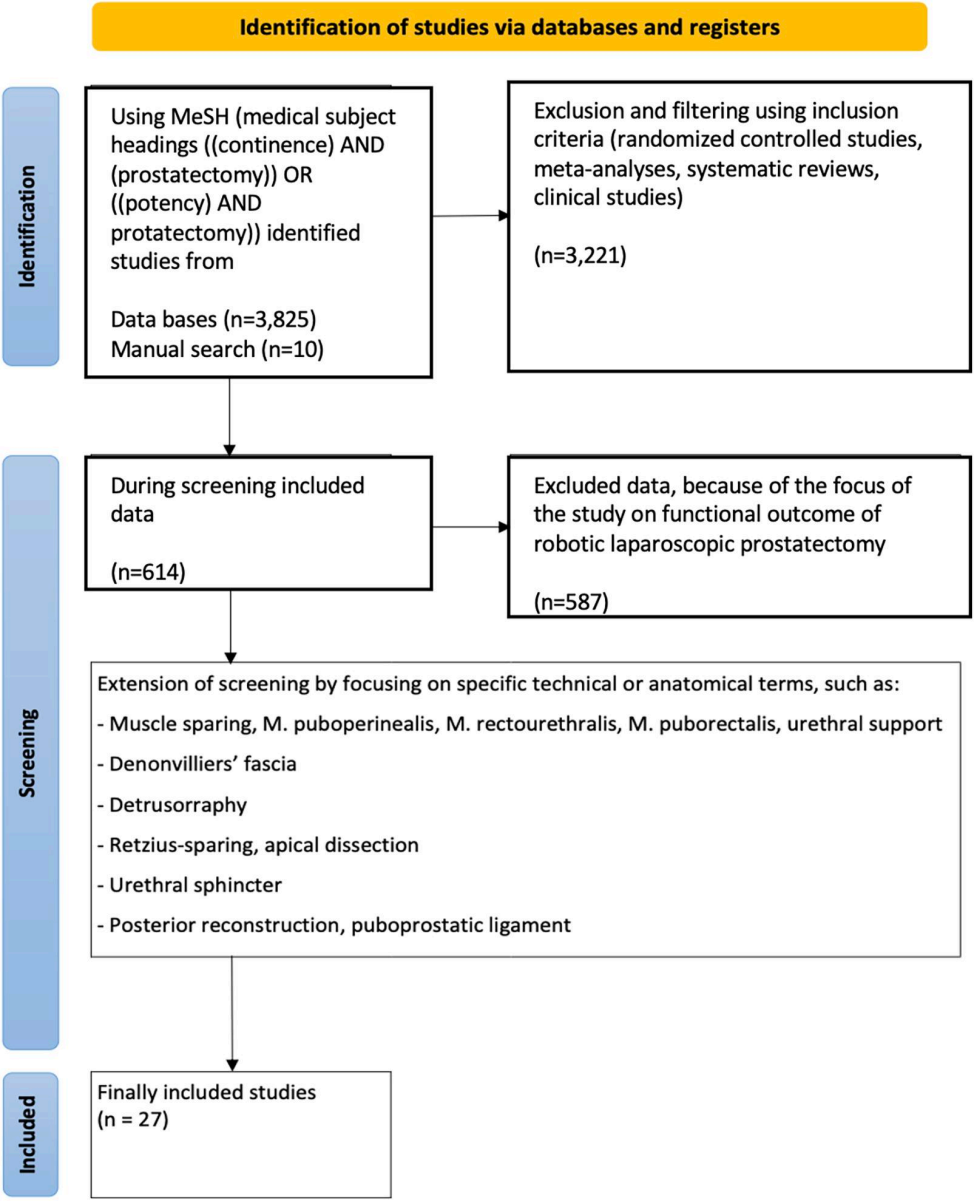
Flowchart of the literature search.

A systematic search strategy was conducted in PubMed using MeSH (Medical Subject Headings) terms [/(continence) AND (prostatectomy)] OR [(potency) AND (prostatectomy)] that yielded 3,825 publications plus 10 articles found by manual search. After applying the inclusion criteria (randomized controlled trials, meta-analyses, reviews, and clinical studies), 614 articles remained. Further selection using the terms “muscle sparing,” “puboperinealis,” “rectourethralis,” “puborectalis”; “urethral support,” “detrusorraphy”; “Denonvillier's fascia,” “Retzius-sparing,” “apical dissection”; “urethral sphincter,” “posterior reconstruction,” “puboprostatic ligament” yielded 27 suitable articles (Figure 1). In the analysis, we focused on continence and positive margins, as the causes of impotence can be multifactorial. However, patient-specific factors must also be considered with respect to continence (8).

Parts of the article are part of a Master Thesis in German (S.S.) at the Danube Private University (see Supplementary Material). Here we used Chat-GPT for translation into English.

## 3 Results

### 3.1 Nomenclature of pelvic anatomy

An exact nomenclature of anatomical structures is important as it allows the surgeon to describe their technique and the specific surgical situation, providing the basis for reproducibility of the technique among colleagues. Generally, a surgical, anatomical, and eponymous terminology can be distinguished (Table 1). The anatomical descriptions are based on the Federative Committee on Anatomical Terminology from 1998 (13). Generally, surgeons describe their technique using “surgical terminology,” translating the classical anatomical nomenclature from Latin (e.g., endopelvic fascia instead of “fascia pelvis parietalis”). Eponymous descriptions are usually based on the name of the first describer (e.g., Plexus Santorini). Different terminologies for the same anatomical structure are often used. The best example is the “paraprostatic or periprostatic fascia” instead of the “levator fascia” or “fascia levatoris ani” (16, 17).

### 3.2 Relevant anatomical structures in radical prostatectomy

**Figure 2:**
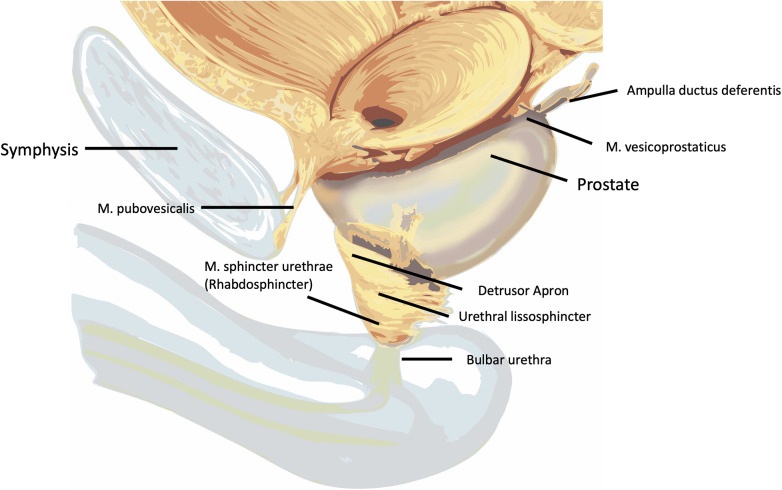
Anatomy of the bladder neck [modified after Dorschner et al. 1994 (21)] with a schematic representation of the key structures relevant to continence.

The prostate is initially covered ventrally by the fascia pelvis parietalis (endopelvic fascia) (14–16). Laterally, a distinction can be made between the fascia levatoris (periprostatic fascia) and the fascia prostatica (prostatic fascia). Posteriorly, the rectovesical septum (Denonvilliers' fascia) covers the prostate and seminal vesicles (18, 19). Ventrally, the prostate is suspended by the puboprostatic ligaments (puboprostatic ligaments) (14, 15). Between these runs the dorsal venous plexus (Plexus Santorini) (14, 15, 21). Laterally on both sides, the arcus tendineus fasciae pelvis (puboprostatic collar) forms further ventral suspension of the urethra (13, 18, 22). Ventrally below the dorsal venous plexus, there is no true prostatic fascia; instead, the anterior fibromuscular stroma and the detrusor apron are located, covered by the fascia pelvis visceralis (13–15, 18, 23) (Table 1; Figure 2).

**Figure 3:**
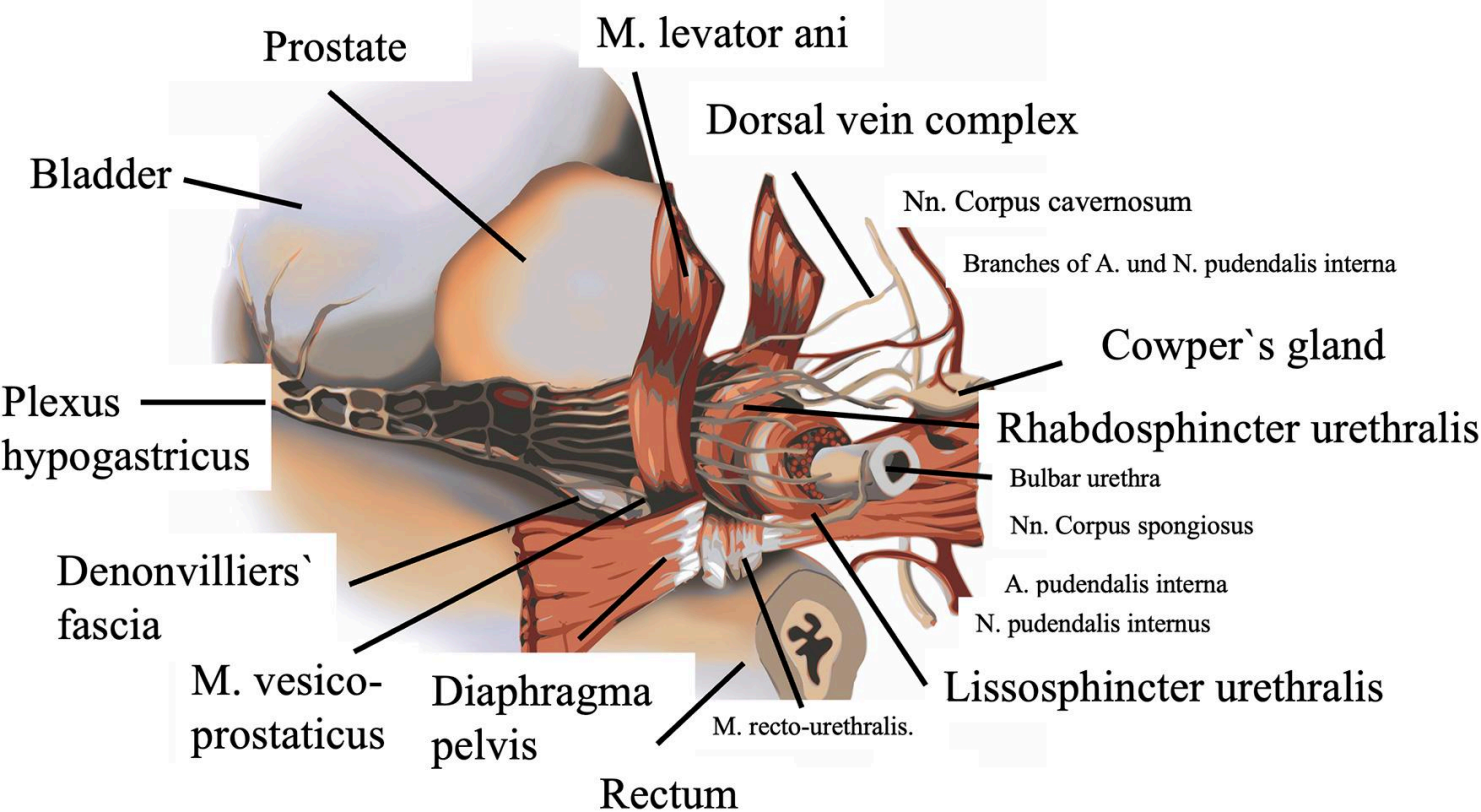
Anatomical details of the urethral sphincter apparatus and its neurovascular supply [modified after Katsimperis et al. 2023 (30)].

**Figure 4:**
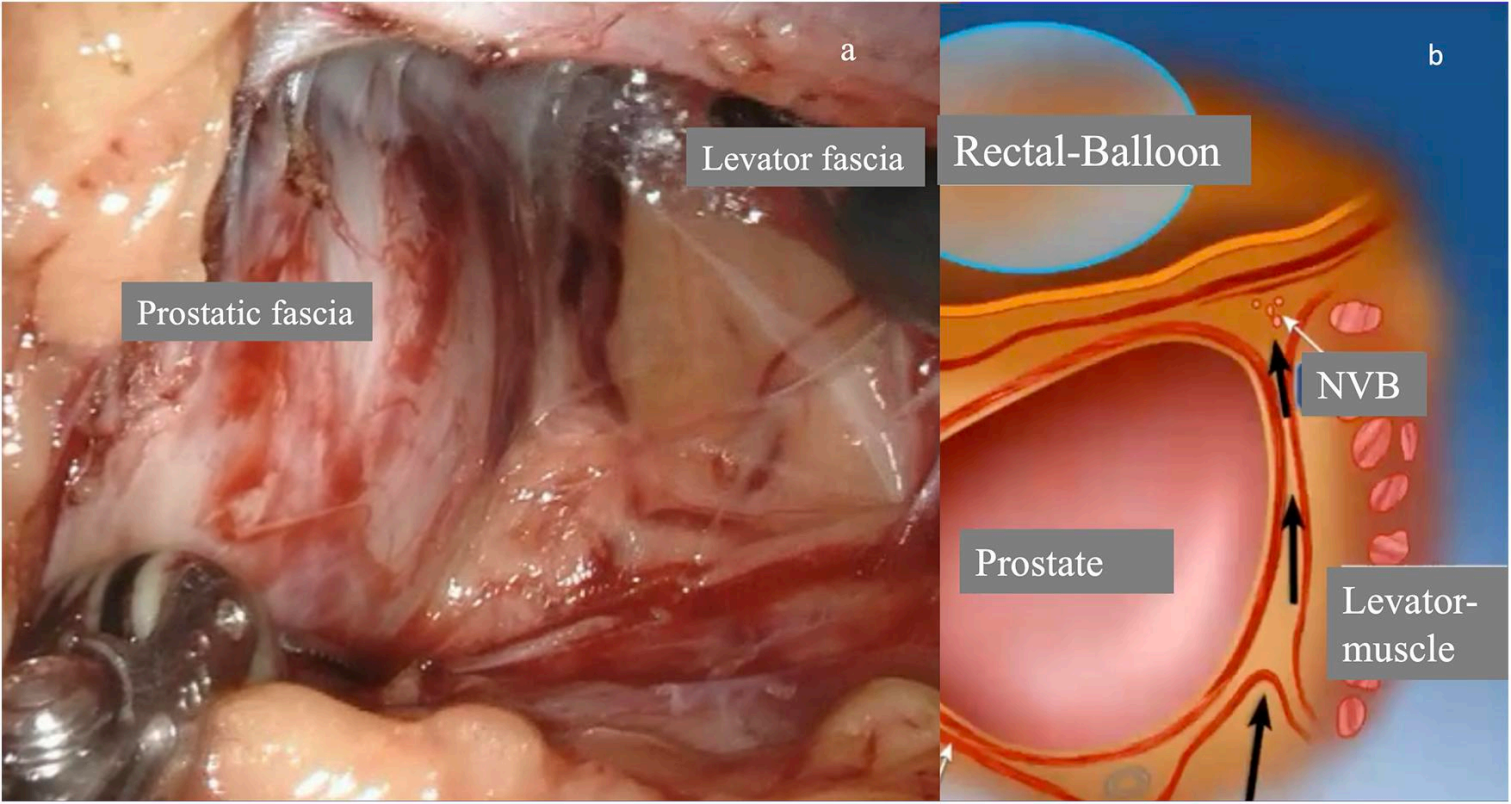
Preservation of the levator fascia as part of the interfascial preservation of the neurovascular bundle. **(a)** Endoscopic view showing the prostate capsule and the preserved fascia of the M. levator ani laterally. **(b)** Schematic representation of the interfascial dissection after opening the endopelvic fascia [modified after Rassweiler et al. 2017 (12)].

Regarding the sphincter apparatus, a primary distinction must be made between the smooth muscle bladder sphincter (M. sphincter vesicae) and the urethral sphincter (M. sphincter urethrae) (18). The urethral sphincter consists of an inner smooth muscle layer (urethral lissosphincter) and the striated outer horseshoe-shaped urethral rhabdosphincter (7, 14, 15, 18). Other essential components of the sphincter apparatus include the smooth muscle M. vesico-prostaticus located dorsal to the bladder neck (18), the ventral extensions of the M. levator ani in the form of the striated M. puboperinealis (18, 21, 24), and the smooth muscle M. recto-urethralis located between the membranous urethra and the rectum (18, 21) (Figures 3, 4).

### 3.3 Physiology of micturition and continence

Continence and micturition are complex processes that are controlled by interactions between the sympathetic and parasympathetic nervous systems (23–27). The structures involved in bladder closure include the M. sphincter vesicalis, also known as the bladder sphincter, and the M. sphincter urethrae, which consists of the smooth muscle inner zone (lissosphincter) and the striated outer, horseshoe-shaped rhabdosphincter (24, 28, 29). The smooth muscle bladder sphincter is innervated by sympathetic fibres and thus contracts involuntarily, while the striated external urethral sphincter is primarily under conscious control. The M. detrusor vesicae, which represents the three-layered bladder muscle, is in a relaxed state during bladder filling and, through its parasympathetically innervated contraction, leads to micturition (27).

### 3.4 Muscular and fascial structures relevant for continence

Muscular and connective tissue components of the pelvic floor play an essential role in continence. The ventral suspension apparatus includes the Mm. pubovesicales, the arcus tendineus fasciae pelvis, and the ligaments puboprostatica and pubovesicale. Dorsally, the Mm. puboperineales and the M. recto-urethralis act as part of the M. levator ani in the sense of a urethral sling mechanism (18, 24–29, 32, 33, 35). The Detrusor apron refers to the smooth muscle extension of the anterior bladder wall that is in direct connection with the pubic bone and wraps around the anterior surface of the prostate in an apron-like manner (28, 29).

### 3.5 Neural structures relevant for continence

The inferior hypogastric plexus, with sympathetic fibres from Th11-L2 and parasympathetic fibres from S2-S4, runs posterior-laterally along the prostate and descends towards the urethra until it passes through the urogenital diaphragm and runs dorsal to the dorsal artery of the penis. It is surgically important that these nerves run below the endopelvic fascia and then between the prostatic and Denonvilliers' fascia before perforating the prostate (26, 29).

The preservation of the neurovascular bundles affects both erectile function and continence postoperatively, which can be attributed to the motor innervation of the urethral sphincter by nerve branches from the dorsal nerve of the penis. Inter- or intra-fascial surgical techniques are suitable for preserving the erectile nerves. In the intra-fascial approach, the goal is to operate below the inner of the two previously described nerve-carrying fasciae, thus directly above the prostate capsule. The inter-fascial approach refers to access between the prostate and levator fasciae (7, 9, 11, 12, 16, 18).

### 3.6 Urethra with sphincter apparatus

After radical prostatectomy, the pseudomembranous part of the urethra retracts, impairing both the function of the urethral sphincter and the urethral closure pressure. It is important to note that the urethral sphincter complex consists not only of the internal bladder sphincter and the external sphincter urethrae, but also includes the pseudomembranous part of the urethra and its surrounding structures summarized as pelvic diaphragm (29). Studies utilizing multiparametric magnetic resonance imaging have shown that the length of the pelvic diaphragm correlates with the functional length of the urethra, which in turn influences sphincter function (39, 40).

### 3.7 Modifications of surgical techniques

The data regarding continence is summarized in Table 2 and presented according to specific techniques. In principle, an extra- and transperitoneal access to the prostate can be distinguished. However, the type of access does not influence the technical modifications described in the literature. Additionally, the Retzius-sparing technique is discussed separately.

**Table 2:** Summary of continence rates of different surgical techniques for radical prostatectomy.

Surgical Technique	Author	Continence rates after Cath. rem. 1 month 3 months 1 year				Comment
Preservation of levator fascia	Kataoka et al. (2023) (31) Laucirica et al. (2020) (40)	n.r. 70,8%	44,6% 83,3%	75,5% 83,3%	78,0% n.r.	Continence = < 2 g/h at pad-test Continence=no pads
Preservation of detrusor apron/detrusorrhaphy	Kucuk et al. (2023) (44) Shin et al. (2019) (45)	25,4% 68,0%	88,9% 86,0%	91,5% n.r.	95,7% 100%	Continence=no pads Continence=no pads
Preservation of pubo-prostatic collar	Kang et al. (2022) (43)	49,2% 20,6%	73,3% 33,3%	86,8% 67,2%	100% 83,0%	Continence=1 safety pad/d Control with anterior suspension
Preservation of urethral Lissosphincter	Almeras et al. (2020) (46) Hoeh et al. (2023) (39)	75,6% n.r. n.r.	82,9% n.r. n.r.	n.r. n.r. n.r.	97,5% 90,5% 63,2%	Continence=no pads Continence=1 Safety pad/d
Bladder-neck sparing	Tunc et al. (2014) (48) Kim et al. (2019) (49)	100% n.r. n.r.	100% n.r. n.r.	n.r. 57,9% 31.8%	n.r. 83,5% 71.2%	Continence=1 Safety pad/d Meta-analysis (*N* = 1880) Control group (*N* = 727)
Retzius-sparing technique	Yee et al. (2022) (55)	33,3% 0,0%	n.r. n.r.	79,2% 66,7%	95,8% 87,5%	Continence=no pads
Posterior reconstruction	Passos et al. (2021) (51)	n.r. n.r.	n.r. n.r.	96,9% 33,3%	n.r. n.r.	Continence=1 Safety pad/d
Anterior und posterior reconstruction	Rinaldi et al. (2023) (52) Puliatti et al. (2019) (53)	n.r. 25,0% 6,4%	33,8% 19,4% 31,3% 25,2%	81,0% 54,3% 56,3% 42,6%	86,3% 64,7% 79,2% 66,0%	Continence=1 Safety pad/d Control (no reconstruction) Continence=1 Safety pad/d Control (posterior reconstruction)

n.r., not reported; Cath. rem, Catheter removal.

#### 3.7.1 Methodology for determining postoperative continence

A critical aspect for evaluating the different surgical techniques is the respective methodology for determining postoperative continence. The studies differ in this regard. Continent status is predominantly evaluated based on questionnaires, often equating the use of no pads (= 0 pad) and the use of a safety pad (= 0–1 pad). Another, somewhat more differentiated evaluation involves quantifying urine loss (in g/h).

In the application of the urine loss ratio (ULR) proposed by Ates et al. (42), which is the weight of urine loss in the pad divided by the daily micturition volume, this is determined during the hospital stay after catheter removal. In our institutional trial, a ULR of less than 0.05 showed an 89% probability that the patient would be continent in 3 months. (Supplementary Table SI; Figure SI).

#### 3.7.2 Preservation of the musculus levator ani and its fascia

The M. puborectalis, together with the M. pubococcygeus and M. iliococcygeus, forms the M. Levator ani. Both Kataoka et al. (31) and Laucirica et al. (40) describe a technique in which the preservation of the Levator ani is essential. Kataoka et al. achieved continence rates of 78.0% after 12 months, while Laucirica et al. achieved even 98.3% after 12 months (31).

Crucial for the functionality of the sphincter complex is the pudendal nerve, which innervates the rhabdosphincter, while the internal sphincter is supplied by sympathetic fibres from the inferior hypogastric plexus. Additionally, the cavernous nerves, which typically arise from the neurovascular bundle, provide direct innervation to the membranous urethra. Preserving the intrapelvic branches of the N. pudendus is particularly suited to the inter-fascial preparation of the prostate, as this preserves the levator fascia and thus keeps the underlying branches intact.

In our own institutional trial, we observed after the inter-fascial technique with preservation of the levator fascia and the puboprostatic collar, and sparing the erectile nerves, a minimal urine loss ratio (ULR) of less than 0.02 was achieved in 92.3% of patients, with a rate of positive margins in stage < pT3 of 7%, in contrast to only 52.3% with sole posterior reconstruction (Supplementary Table SI; Figure SI).

#### 3.7.3 Preservation of the dorsal venous complex (DVC) and puboprostatic collar

**Figure 5:**
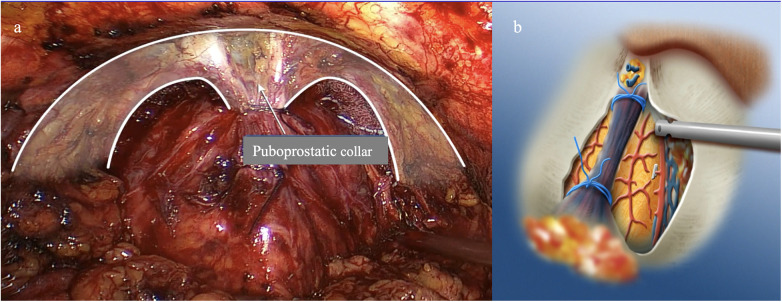
Preservation of the puboprostatic collar with proximal transection of the dorsal venous plexus and only partial incision of the puboprostatic ligaments. **(a)** Endoscopic image after proximal transection of the dorsal venous plexus near the bladder neck. **(b)** Schematic representation of the preservation of the components of the ventral sphincter apparatus [modified after Rassweiler et al. 2017 (12)].

The Santorini Plexus, or dorsal venous complex (DVC), is anchored below the symphysis. By performing a proximal bypass and transecting the DVC at the basal end of the prostate while dissecting towards the apex, one can largely preserve the puboprostatic ligaments along with the surrounding fibromuscular tissue (Figure 5). Since this resembles a shirt collar, it is also referred to as the puboprostatic collar (22).

In the course of a modified apical dissection, attempts are made to preserve the puboprostatic ligaments as well as the apical portion of the endopelvic fascia (41, 43). Kang et al. (43) demonstrated that this apical dissection technique led to significantly better early continence after 3 months (73% vs. 33%) compared to the anterior suspension suture between the DVC and the symphysis, while after 9 months, no significant difference remained (96.6% vs. 81%). The rate of positive margins in stage < pT3 was similar in both groups (9% vs. 8%).

#### 3.7.4 Preservation of the detrusor apron/detrusorrhaphy

**Figure 6:**
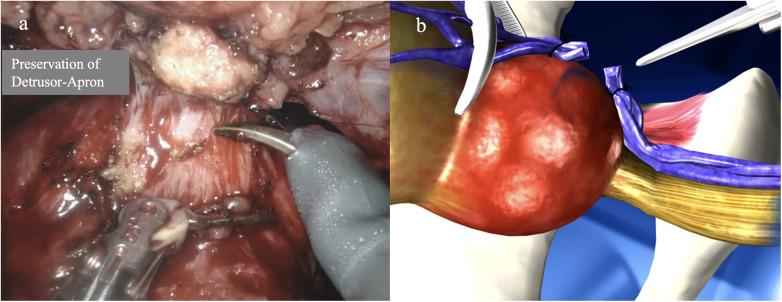
Preservation of the detrusor apron after transection of the dorsal venous plexus. **(a)** Endoscopic image with sharp dissection on the anterior surface of the prostate. **(b)** Schematic representation of the dissection technique.

The “ultra-dissection technique” is a completely antegrade approach (44): After incision of the peritoneum and dissection of the seminal vesicles, the Denonvilliers' fascia is incised posteriorly. Only then is access obtained to the Retzius space and the bladder neck. After incision of the bladder neck with access to the already dissected urogenital space, the prostatic vascular pillars are controlled, and then the endopelvic fascia is preserved. Once reaching the medial part of the prostatic capsule, the DVC is ligated. Through blunt dissection beneath the detrusor apron, efforts are made to preserve it before transecting the urethra (Figure 6). Kucuk et al. (44) achieved a continence rate of 95.7% after 12 months with this technique, with 6.5% of patients having an R1 situation in < pT3 stages. The detrusorrhaphy-technique proposed by Shin and Lee (45) reinforces the detrusor apron which resulted to an 86% continence rate after 1 month and 100% continence after 12 months (Table 2).

#### 3.7.5 Preservation of the distal smooth muscle urethra (lissosphincter)

**Figure 7:**
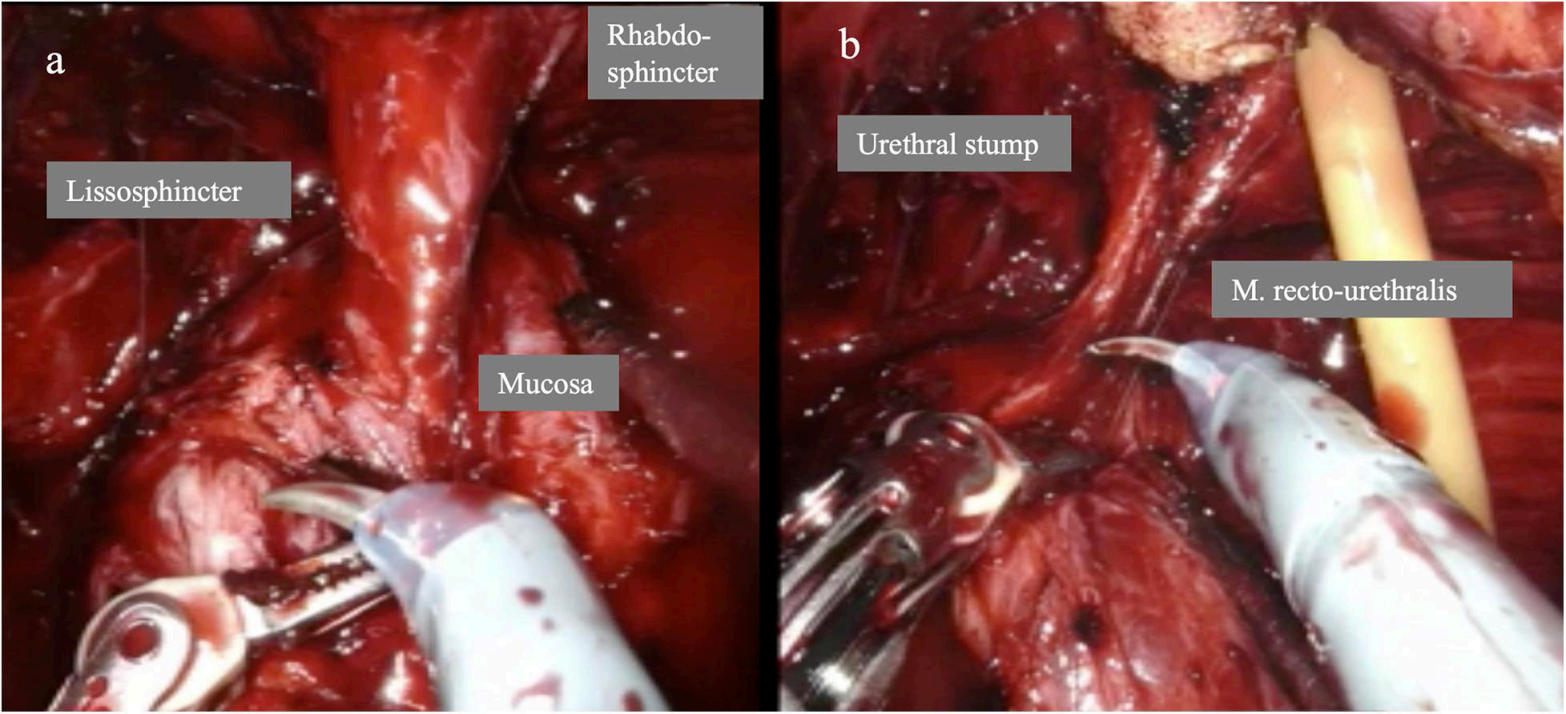
Maximum preservation of the functional urethra and retention of the M. recto-urethralis. **(a)** Endoscopic view of the urethral rhabdosphincter, lissosphincter, and the mucosa immediately at the edge (“nodge”) of the prostate.**(b)** Endoscopic image after transection of the urethra with a long urethral stump and preservation of the M. recto-urethralis.

Arroyo et al. (34) recommend a posterior-to-anterior transection of the urethra, using the seminal colliculus as an anatomical landmark, to preserve the Lissosphincter and its autonomic innervation while ensuring adequate distance between the apex of the prostate and the rhabdosphincter. In this process, care is taken to identify the mucosa to subsequently locate and preserve the M. recto-urethralis posteriorly (Figure 7). This is important for the posterior reconstruction, which consists of anastomosis between the vesico-prostatic muscle and the recto-urethral muscle (45). Hoeh et al. (39) compared maximum preservation (FFLU=full functional length of urethra) with a historical control group without FFLU. After 450 days, 90.5% of the first group were continent compared to only 63.2% in the control group.

#### 3.7.6 Preservation of the bladder neck

Some authors suggest that preserving the bladder neck has a positive effect on continence (46–49). After coagulating superficial bladder vessels, the zone between the bladder neck and prostate, which consists of fatty connective tissue (46, 47), is identified. This can provide direct visualization of the urethra and the muscle fibres of the internal sphincter through blunt dissection, facilitating the transection of the urethra. By preserving the bladder neck, Tunc et al. (48) achieved absolute continence in 100% of their patients. In the meta-analysis by Kim et al. (49), a 1-year continence rate of 83.5% was observed compared to 71.2% in the control group.

#### 3.7.7 Opening of the bladder neck and access to the seminal vesicles

**Figure 8:**
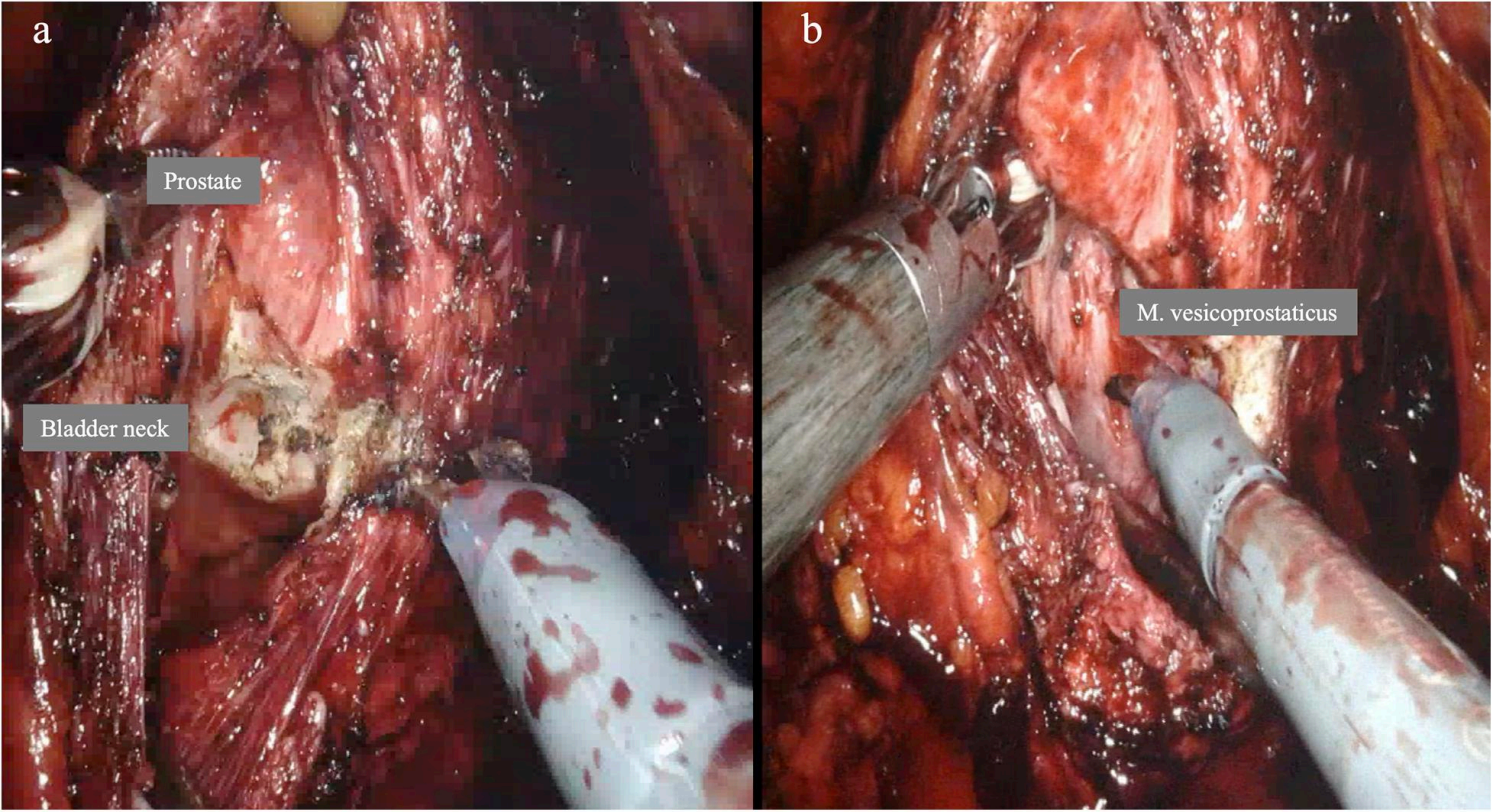
Transection of the dorsal bladder neck and the M. vesico-prostaticus with access to the urogenital space (retrovesical) with the vas deferens and seminal vesicles. **(a)** Transection of the transversely oriented detrusor distal using HF electro-incision. **(b)** Representation and incision of the M. vesico-prostaticus.

After identifying the ostia, the prostate is separated from the M. detrusor vesicae. The M. vesico-prostaticus is then divided, and access is created to the urogenital space (retrovesical) with the seminal vesicles and ductus deferentes (Figure 8). When controlling the prostatic pillars, it is crucial to ensure that this is not done too proximally, as this can lead to injury to the branches of the hypogastric plexus. We prefer to use Hem-o-lock clips here to minimize operative thermal trauma.

#### 3.7.8 Posterior reconstruction

**Figure 9:**
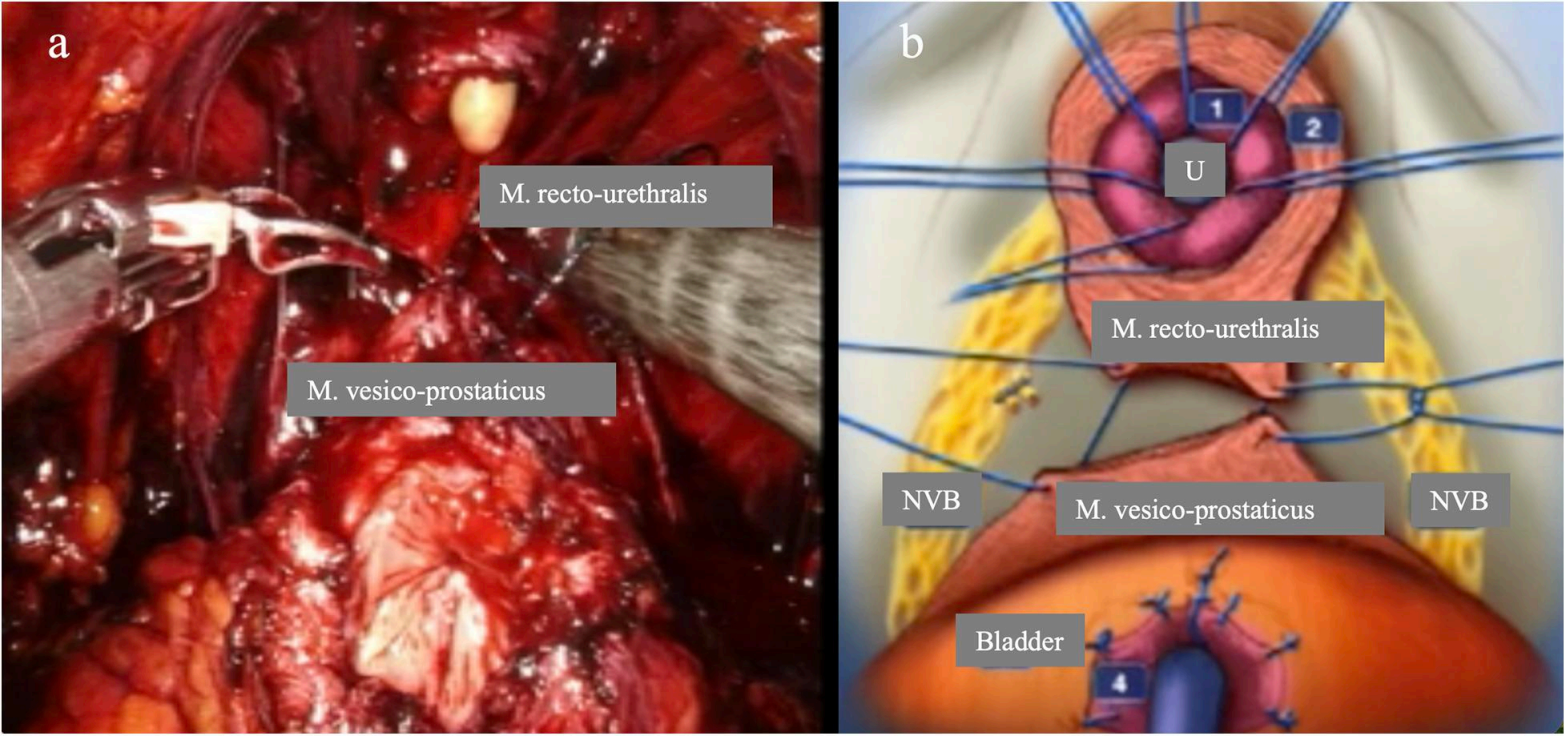
Posterior reconstruction according to Rocco 2009 (50). **(a)** Endoscopic image of the suture between the M. vesico-prostaticus and M. recto-urethralis. **(b)** Schematic representation [modified after Rocco 2009 (50)].

In the posterior reconstruction according to Rocco (50), the cranial stump of the M. vesico-prostaticus is sutured to the stump of the recto-urethralis muscle to strengthen the posterior sphincter apparatus (Figure 9). Paraurethral remnants of Denonvilliers' fascia can also be used for this purpose (34, 46). Rocco et al. (50) achieved a 3-month continence rate of 92.3% compared to 76.9% in the control group. Passos et al. (51) confirmed these results (96.9% vs. 33.3%).

#### 3.7.9 Complete reconstruction

The combination of the aforementioned procedure with the anterior anchoring of the vesicourethral anastomosis at the puboprostatic ligament is intended to ensure a tighter anterior and posterior vesicourethral angle and thus contribute to continence preservation. For this, the anterior part of the detrusor apron and the puboprostatic ligament are reconstructed (51, 52). Rinaldi et al. (52) observed a continence rate of 86.3% after one year compared to 64.7% in the control group (only posterior reconstruction). In the study by Puliatti et al. (53), a significant advantage was shown after 6 months (72.9% vs. 51.1%), while after 1 year, no significant difference remained (79.2% vs. 66%).

#### 3.7.10 Preservation of the retropubic space (retzius space)

In 2010, Bocciardi and his team introduced the Retzius-sparing method (54). This technique preserves nearly all anterior fascial structures of the pelvis (endopelvic fascia, levator fascia, detrusor apron, dorsal venous complex, and the puboprostatic ligaments). One begins with the incision of the parietal peritoneum at the level of the vesico-rectal pouch and resects the prostate and seminal vesicles beneath the endopelvic fascia (54–57). According to Yee et al. (55), patients in whom the Retzius space was preserved showed a 3-month continence rate of 79.2% compared to 66.7% in the control.

## 4 Discussion

Incontinence is one of the most burdensome complications of radical prostatectomy, as it involves resection of critical functional structures of the sphincter apparatus. This affects not only the prostate itself but also the smooth muscle unit and neural supply of the urethral sphincter, upon which postoperative continence essentially depends (58). Urodynamically, intrinsic sphincter insufficiency has been demonstrated in 40%–80% of patients after open radical prostatectomy (59).

Since the introduction of robot-assisted laparoscopic radical prostatectomy (RALP), a primary focus has been on improving postoperative continence, with ongoing investigations into anatomy and numerous technical modifications being proposed (Table 2). Undoubtedly, significant progress has been made in this area, though a current technique of RALP should not focus solely on a single detail but rather represent a consecutive application of the proposed surgical steps. The following techniques appear particularly important.

### 4.1 Preservation of the M. levator ani with the Levator Fascia

The preservation of the levator fascia occurs almost automatically with intra- and interfascial protection of the neurovascular bundles: one remains in a layer that, aside from apical perforating fibres of the levator ani, contains no muscular structures. Additionally, the intrapelvic branch of the pudendal nerve, which innervates the external urethral sphincter (urethral rhabdosphincter), runs beneath the levator fascia and is crucial for postoperative continence (26). Locally advanced tumors that have already perforated the prostatic fascia, particularly apically, pose a challenge for preserving the levator fascia. These can only be resected extra-fascially, meaning with the levator fascia included. Nevertheless, we feel that every effort should be made to preserve as much of the M. levator ani as possible.

### 4.2 Preservation of the puboprostatic collar

In the course of a modified apical dissection, efforts are made to preserve the puboprostatic ligaments as well as the apical portion of the endopelvic fascia along with the detrusor apron (6). Ultimately, in this technique, the apical attachment of the M. pubococcygeus is divided to visualize the apex of the prostate and the dorsal vein complex (DVC) laterally, allowing for distal rather than proximal resection of the DVC (Figures 5, 6). Kang et al. (43) showed that this eliminates the need for a suspension suture. Moreover, Shin et al. (45) showed that a further reinforcement of the detrusor apron might be beneficial to achieve early continence. However, according to our experience, this is not necessary.

### 4.3 Preservation of the urethral lissosphincter

Numerous authors emphasize that preserving the urethral lissosphincter, corresponding to maximum retention of the functional smooth muscle sphincter while simultaneously protecting the rhabdosphincter, is crucial for good postoperative continence (37–39). The seminal colliculus represents the distal boundary (Figure 7). It is important to understand the anatomical and functional importance of this structure. The urethral lissosphincter is completely different from the so called “inner sphincter” created by detrusor muscle at the bladder neck (47, 48). Bladder neck preservation cannot be accomplished in all cases (i.e., in cases with a larger medium lobe of the prostate), however, the urethral lissosphincter can be and should be preserved in all cases.

### 4.4 Preservation of the neurovascular bundle

Preservation of the neurovascular bundle also positively influences postoperative continence. However, this must always be considered in conjunction with the other surgical steps, as the outcomes for groups with and without nerve preservation differ significantly (36). Nonetheless, Reeves et al. (60) demonstrated in a meta-analysis of 13,749 patients in 27 studies that this benefit only applies to early continence within the first 6 months. After that, no significant advantage remained.

### 4.5 Preservation of the bladder neck

Tunc et al. (48) demonstrated their bladder neck-preserving technique, with all 54 patients being continent after catheter removal. There was no change in continence even after one month. In 82.6% of patients, the neurovascular bundles could be preserved on one or both sides. However, there is a lack of data regarding the exact definition of continence. The meta-analysis by Kim et al. (49) showed a 1-year continence advantage of 83.5% compared to 71.2%. By preserving the bladder neck, unnecessary damage to the detrusor muscle is prevented. However, as mentioned already, there are technical limitations to preserving the bladder neck, especially in cases with pronounced median lobe involvement (47).

### 4.6 Posterior reconstruction

Rocco et al. (50) highlighted in their 2007 prospective study the positive influence of posterior reconstruction on continence (Figure 9). Kataoka et al. (31) emphasized in their study the importance of the M. puboperinealis and M. rectourethralis, as these stabilize the urethra. The puboperinealis muscle has been described as responsible for the quick-stop phenomenon of urination in men (25), while the recto-urethralis muscle fills part of the mid-space between the right and left puboperinealis muscles, thereby contributing to the stability of the pelvic floor and continence. There are further modifications in the literature, i.e., by taking a part of the Denonvilliers` fascia (51). However, we emphasize, that surgeons should stick to the original technique (Figure 9).

### 4.7 Anterior and posterior reconstruction

The studies by Rinaldi et al. (52) and Puliatti et al. (53) from 2019 demonstrate that additional anterior reconstruction mainly positively affects early continence. In transperitoneal procedures, this can be combined with a suture of the bladder peritoneum to the stump of the dorsal vein complex to prevent lymphoceles (61).

### 4.8 Preservation of the retzius space

This is certainly a technically demanding technique aimed at preserving significantly anterior structures of the sphincter apparatus (54–57). Two meta-analyses indicate that this leads to improved early continence rates but is also associated with a higher rate of positive margins (55, 56). The learning curve plays a crucial role here as well. Interestingly, the difference did not reach significance in ≥ T3 tumours (56). We still see only a selective indication for preserving the Retzius space, as tumour-free margins should remain the primary goal. Recently, attempts have also been made in the context of the classical descending approach to preserve as many anterior structures as possible with the “Hood Technique,” which also observed very good early continence (83% after 1 month) (62).

### 4.10 Limitation of the article

Even if the selection of articles was based on a systematic selection using MeSH (Figure 1) it was necessary also to include articles of lower quality, just describing early experiences when applying a new surgical step or modifying their previous technique. Not every surgical step has been tested in a randomized controlled trial. Moreover, the definition of continence and the endpoints vary across the different studies. Therefore, we focused on this on specific column in Table 2. Due to the small numbers of studies describing different surgical steps, it was not possible to stratify into different subgroups or to create any Forrest plots. As mentioned earlier, the main focus of this article is educational (63, 64).

## 5 Conclusions

The increase of knowledge of the video-anatomical details of the prostate and surrounding structures allows translation into novel surgical techniques of RALP (6, 7, 18, 63, 64). Based on the analysis of the literature and own institutional experiences the following surgical details proved to be significant:

- Preservation of the levator fascia and M. levator ani
- Preservation of the puboprostatic collar
- Long urethral stump with preservation of the lissosphincter
- Preservation of the bladder neck, if possible
- Posterior reconstruction between the M. vesico-prostaticus and M. rectourethralis
- Individual techniques, such as the Retzius-sparing technique, showed lower incontinence rates but appear to be less secure regarding tumour-free margins.

Finally, individual patient-specific factors (age, BMI, tumour localization, functional urethral length) must enable an adequate implementation of the mentioned surgical techniques.

## Data Availability

The original contributions presented in the study are included in the article/Supplementary Material, further inquiries can be directed to the corresponding author.

## Supplementary material

Supplementary Figure S1 Institutional comparison of continence development after robot-assisted laparoscopic radical prostatectomy (RALP) with preservation of the levator fascia, retention of the puboprostatic collar and urethral lissosphincter, as well as posterior reconstruction (new technique) compared to the previously used “old technique”.
