# Supplementary figures and images for: Demonstration of anatomical and technical details of robotic laparoscopic radical prostatectomy as described in the current literature
Source: Front Surg. 2026 May 7;13:1804051. doi: 10.3389/fsurg.2026.1804051 (PMC13189793; doi:10.3389/fsurg.2026.1804051)

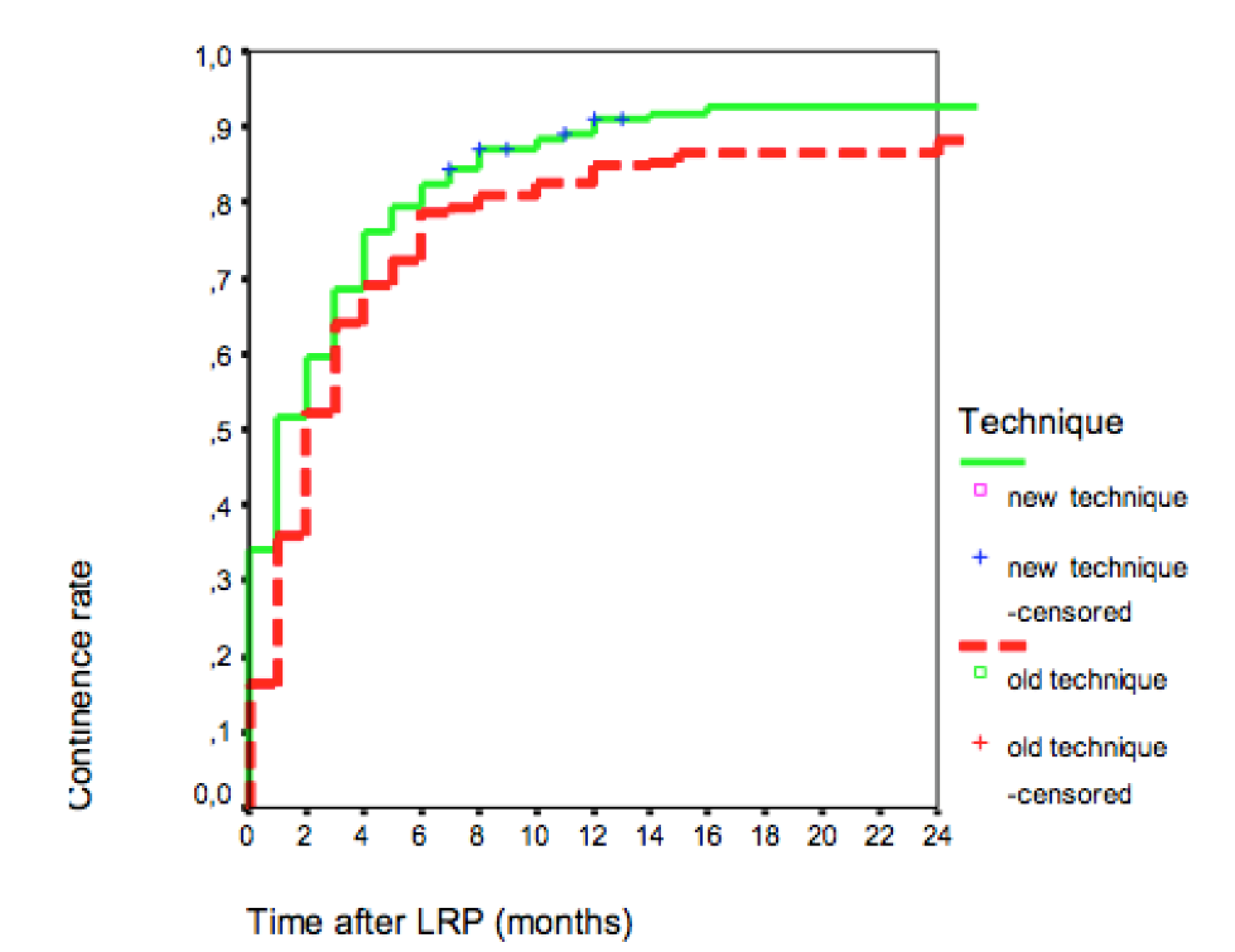

Supplement: Supplementary Figure S1 — Institutional comparison of continence development after robot-assisted laparoscopic radical prostatectomy (RALP) with preservation of the levator fascia, retention of the puboprostatic collar and urethral lissosphincter, as well as posterior reconstruction (new technique) compared to the previously used “old technique”. [file Image1.tiff]
