# Supplementary material for: Demonstration of anatomical and technical details of robotic laparoscopic radical prostatectomy as described in the current literature
Source: Front Surg. 2026 May 7;13:1804051. doi: 10.3389/fsurg.2026.1804051 (PMC13189793; doi:10.3389/fsurg.2026.1804051)
Supplement: Supplementary file 2 [file Table1.docx]

**Supplemental Table I:** Impact of preservation of levator fascia and puboprostatic collar together with posterior reconstruction on early continence: same surgeon (J.R.), < 70 years, pT2-3, bilateral nerve-sparing (modified from Rassweiler & Sander 2025^1^).

| **Urine Loss Ratio (ULR)** | **No posterior reconstruction**  **No preservation of pubo-prostatic collar**  **No preservation of levator fascia** | **Posterior reconstruction**  **No preservation of pubo-prostatic collar**  **No preservation of levator fascia** | **Posterior reconstruction**  **Preservation of pubo-prostatic collar**  **Preservation of levator fascia** |
| --- | --- | --- | --- |
| <0.02 | 55.2% | 52.3% | 92.3% |
| <0.05 | 22.7% | 18.2% | 7.7% |
| <0.11 | 9.4% | 18.2% | - |
| <0.16 | 6.4% | 9.1% | - |
| **Continent**  (0-1 safety pads after 3 months) | **77.9%** | **70,5%** | **100.0%** |

**ULR = Urin-loss in pads / daily micturation volume following removal of the catheter (Ates et al. 2007^42^)**

1. Rassweiler J, Sander S (2025): Operative Techniken zur Verbesserung der Kontinenz nach laparoskopischer roboterassistierter Prostatektomie anhand von videoanatomischen Strukturen – eine Übersichtsarbeit. (english abstract). Die Urologie published on-line (https://doi.org/10.1007/s00120-025-02627-0)
